# Supplementary material for: Modeling glioblastoma heterogeneity as a dynamic network of cell states
Source: Mol Syst Biol. 2021 Sep 16;17(9):e10105. doi: 10.15252/msb.202010105 (PMC8444284; doi:10.15252/msb.202010105)
Supplement: Supplementary file 6 — Source Data for Figure 5 [file MSB-17-e10105-s004.zip › Figure5A_sourcedata/GSEA_3017/nosigdb_stateF.GseaPreranked.1622795015644/FELDMAN_2020_S.html]

Details for gene set FELDMAN\_2020\_S[GSEA]

|  || Dataset | state33017 |
| Phenotype | NoPhenotypeAvailable |
| Upregulated in class | na\_neg |
| GeneSet | FELDMAN\_2020\_S |
| Enrichment Score (ES) | -0.629883 |
| Normalized Enrichment Score (NES) | -4.22492 |
| Nominal p-value | 0.0 |
| FDR q-value | 0.0 |
| FWER p-Value | 0.0 |
Table: GSEA Results Summary

  

Fig 1: Enrichment plot: FELDMAN\_2020\_S      
 Profile of the Running ES Score & Positions of GeneSet Members on the Rank Ordered List

  

| PROBE | GENE SYMBOL | GENE\_TITLE | RANK IN GENE LIST | RANK METRIC SCORE | RUNNING ES | CORE ENRICHMENT || 1 | CCL2 |  |  | 7 | 1.774 | 0.0217 | No |
| 2 | FTL |  |  | 32 | 1.036 | 0.0251 | No |
| 3 | RPS27L |  |  | 33 | 1.025 | 0.0395 | No |
| 4 | SAT1 |  |  | 45 | 0.957 | 0.0478 | No |
| 5 | SESN3 |  |  | 56 | 0.908 | 0.0560 | No |
| 6 | MALAT1 |  |  | 90 | 0.773 | 0.0514 | No |
| 7 | DDIT4 |  |  | 216 | 0.579 | 0.0013 | No |
| 8 | BTG1 |  |  | 222 | 0.574 | 0.0071 | No |
| 9 | BEX4 |  |  | 242 | 0.559 | 0.0061 | No |
| 10 | ARL6IP5 |  |  | 264 | 0.542 | 0.0039 | No |
| 11 | CCNG1 |  |  | 292 | 0.515 | -0.0014 | No |
| 12 | EEF1A1 |  |  | 417 | 0.464 | -0.0527 | No |
| 13 | TSC22D1 |  |  | 473 | 0.443 | -0.0721 | No |
| 14 | YPEL5 |  |  | 583 | 0.403 | -0.1172 | No |
| 15 | PAIP2 |  |  | 739 | 0.362 | -0.1843 | No |
| 16 | HES4 |  |  | 950 | 0.317 | -0.2777 | No |
| 17 | SCRG1 |  |  | 1032 | 0.304 | -0.3112 | No |
| 18 | LYPD1 |  |  | 1094 | 0.293 | -0.3355 | No |
| 19 | RPA2 |  |  | 1490 | -0.256 | -0.5160 | No |
| 20 | ACAT2 |  |  | 1512 | -0.263 | -0.5220 | No |
| 21 | MSH6 |  |  | 1517 | -0.264 | -0.5202 | No |
| 22 | TMEM97 |  |  | 1629 | -0.293 | -0.5678 | No |
| 23 | EIF4EBP1 |  |  | 1681 | -0.312 | -0.5872 | No |
| 24 | PHLDA1 |  |  | 1718 | -0.324 | -0.5994 | No |
| 25 | RAD51 |  |  | 1723 | -0.326 | -0.5967 | No |
| 26 | MCM3 |  |  | 1738 | -0.335 | -0.5985 | No |
| 27 | EXOSC9 |  |  | 1746 | -0.338 | -0.5970 | No |
| 28 | RAB13 |  |  | 1761 | -0.345 | -0.5987 | No |
| 29 | RAN |  |  | 1772 | -0.350 | -0.5984 | No |
| 30 | LIMS1 |  |  | 1807 | -0.365 | -0.6091 | No |
| 31 | CDC45 |  |  | 1836 | -0.382 | -0.6168 | No |
| 32 | CDCA4 |  |  | 1852 | -0.391 | -0.6183 | No |
| 33 | EXOSC8 |  |  | 1865 | -0.397 | -0.6183 | No |
| 34 | RFC4 |  |  | 1887 | -0.413 | -0.6223 | No |
| 35 | ASF1B |  |  | 1897 | -0.419 | -0.6206 | No |
| 36 | POLD3 |  |  | 1918 | -0.436 | -0.6237 | Yes |
| 37 | GGCT |  |  | 1930 | -0.442 | -0.6227 | Yes |
| 38 | GINS2 |  |  | 1932 | -0.445 | -0.6169 | Yes |
| 39 | SNRPB |  |  | 1937 | -0.447 | -0.6125 | Yes |
| 40 | RFC2 |  |  | 1954 | -0.457 | -0.6135 | Yes |
| 41 | EIF1AX |  |  | 1959 | -0.458 | -0.6089 | Yes |
| 42 | DNMT1 |  |  | 1969 | -0.465 | -0.6066 | Yes |
| 43 | C19orf48 |  |  | 1979 | -0.474 | -0.6041 | Yes |
| 44 | NASP |  |  | 1988 | -0.481 | -0.6010 | Yes |
| 45 | PGP |  |  | 1990 | -0.482 | -0.5947 | Yes |
| 46 | CDC6 |  |  | 1991 | -0.483 | -0.5879 | Yes |
| 47 | MCM4 |  |  | 1993 | -0.487 | -0.5815 | Yes |
| 48 | ARL6IP1 |  |  | 2001 | -0.494 | -0.5779 | Yes |
| 49 | TK1 |  |  | 2003 | -0.497 | -0.5713 | Yes |
| 50 | SIVA1 |  |  | 2014 | -0.505 | -0.5689 | Yes |
| 51 | MAGOHB |  |  | 2015 | -0.505 | -0.5618 | Yes |
| 52 | FEN1 |  |  | 2018 | -0.511 | -0.5555 | Yes |
| 53 | RANBP1 |  |  | 2024 | -0.517 | -0.5506 | Yes |
| 54 | HAUS8 |  |  | 2026 | -0.517 | -0.5438 | Yes |
| 55 | CACYBP |  |  | 2028 | -0.518 | -0.5369 | Yes |
| 56 | HELLS |  |  | 2038 | -0.528 | -0.5337 | Yes |
| 57 | CDCA3 |  |  | 2041 | -0.529 | -0.5272 | Yes |
| 58 | NCL |  |  | 2045 | -0.532 | -0.5211 | Yes |
| 59 | SRSF7 |  |  | 2056 | -0.544 | -0.5181 | Yes |
| 60 | CTNNAL1 |  |  | 2059 | -0.548 | -0.5114 | Yes |
| 61 | PTMA |  |  | 2065 | -0.570 | -0.5057 | Yes |
| 62 | ZWINT |  |  | 2069 | -0.577 | -0.4990 | Yes |
| 63 | CENPA |  |  | 2073 | -0.586 | -0.4921 | Yes |
| 64 | CENPK |  |  | 2082 | -0.607 | -0.4873 | Yes |
| 65 | USP1 |  |  | 2084 | -0.612 | -0.4792 | Yes |
| 66 | FAM111B |  |  | 2094 | -0.637 | -0.4744 | Yes |
| 67 | MIS18A |  |  | 2096 | -0.639 | -0.4659 | Yes |
| 68 | DNAJC9 |  |  | 2097 | -0.641 | -0.4568 | Yes |
| 69 | RFC3 |  |  | 2098 | -0.642 | -0.4478 | Yes |
| 70 | BRCA2 |  |  | 2100 | -0.650 | -0.4391 | Yes |
| 71 | SNRPD1 |  |  | 2105 | -0.653 | -0.4318 | Yes |
| 72 | PSMC3IP |  |  | 2106 | -0.659 | -0.4226 | Yes |
| 73 | AURKA |  |  | 2110 | -0.673 | -0.4145 | Yes |
| 74 | RPA3 |  |  | 2112 | -0.678 | -0.4054 | Yes |
| 75 | ANP32B |  |  | 2119 | -0.700 | -0.3984 | Yes |
| 76 | SRSF2 |  |  | 2122 | -0.706 | -0.3894 | Yes |
| 77 | PA2G4 |  |  | 2123 | -0.707 | -0.3794 | Yes |
| 78 | CENPM |  |  | 2125 | -0.709 | -0.3699 | Yes |
| 79 | HNRNPAB |  |  | 2130 | -0.729 | -0.3615 | Yes |
| 80 | ATAD5 |  |  | 2133 | -0.742 | -0.3520 | Yes |
| 81 | VRK1 |  |  | 2134 | -0.743 | -0.3416 | Yes |
| 82 | BRCA1 |  |  | 2138 | -0.758 | -0.3323 | Yes |
| 83 | FBXO5 |  |  | 2144 | -0.779 | -0.3237 | Yes |
| 84 | GMNN |  |  | 2146 | -0.785 | -0.3131 | Yes |
| 85 | SMC3 |  |  | 2150 | -0.800 | -0.3033 | Yes |
| 86 | DEK |  |  | 2154 | -0.817 | -0.2932 | Yes |
| 87 | DUT |  |  | 2155 | -0.824 | -0.2816 | Yes |
| 88 | MCM7 |  |  | 2160 | -0.839 | -0.2716 | Yes |
| 89 | MCM10 |  |  | 2169 | -0.858 | -0.2633 | Yes |
| 90 | CCDC34 |  |  | 2180 | -0.898 | -0.2553 | Yes |
| 91 | CHAF1A |  |  | 2182 | -0.903 | -0.2431 | Yes |
| 92 | ORC6 |  |  | 2188 | -0.937 | -0.2322 | Yes |
| 93 | LMNB1 |  |  | 2194 | -0.950 | -0.2212 | Yes |
| 94 | UBE2T |  |  | 2196 | -0.965 | -0.2081 | Yes |
| 95 | CCNB2 |  |  | 2200 | -0.983 | -0.1957 | Yes |
| 96 | ESCO2 |  |  | 2204 | -1.004 | -0.1830 | Yes |
| 97 | SMC2 |  |  | 2205 | -1.006 | -0.1688 | Yes |
| 98 | DHFR |  |  | 2212 | -1.040 | -0.1570 | Yes |
| 99 | CLSPN |  |  | 2216 | -1.060 | -0.1434 | Yes |
| 100 | CENPU |  |  | 2219 | -1.088 | -0.1291 | Yes |
| 101 | H2AFZ |  |  | 2220 | -1.090 | -0.1137 | Yes |
| 102 | CDK1 |  |  | 2225 | -1.153 | -0.0994 | Yes |
| 103 | PTTG1 |  |  | 2226 | -1.198 | -0.0825 | Yes |
| 104 | MAD2L1 |  |  | 2228 | -1.204 | -0.0661 | Yes |
| 105 | RRM2 |  |  | 2234 | -1.268 | -0.0505 | Yes |
| 106 | DLGAP5 |  |  | 2235 | -1.273 | -0.0326 | Yes |
| 107 | ATAD2 |  |  | 2238 | -1.381 | -0.0141 | Yes |
| 108 | HMGB2 |  |  | 2241 | -1.469 | 0.0056 | Yes |
Table: GSEA details [plain text format]

  

Fig 2: FELDMAN\_2020\_S: Random ES distribution      
 Gene set null distribution of ES for **FELDMAN\_2020\_S**
